# Revisiting Härtel’s technique for percutaneous transoval glycerol injection

**DOI:** 10.1007/s00701-025-06526-3

**Published:** 2025-04-30

**Authors:** Haldor Slettebø, Tomas Sakinis

**Affiliations:** 1 Department of Neurosurgery, Oslo University Hospital – Rikshospitalet, Oslo, Norway; 2 Department of Radiology, Oslo University Hospital – Rikshospitalet, Oslo, Norway; 3 Institute of Clinical Medicine, Faculty of Medicine, University of Oslo, Oslo, Norway

**Keywords:** Meckel’s cave, Härtel’s technique, Glycerol injection, Trigeminal neuralgia, Imaging-based simulation

## Abstract

**Purpose:**

Percutaneous transoval glycerol injection (GI) has been widely used since 1981 in the treatment of patients with trigeminal neuralgia. However, outcomes have been more variable than with other percutaneous treatments. Although most authors state that they use Härtel’s technique, the variations are numerous—which may explain procedural problems and most of the poor results. The aim of the present imaging-based study, therefore, was to revisit Härtel’s technique and identify optimal landmarks for guiding the needle from the cheek to Meckel’s cave.

**Methods:**

Eleven patients referred for trigeminal neuralgia were studied. We used CT- and MRI-based simulations to determine the optimal entry points in the cheek and trajectories through foramen ovale (FO) to reach Meckel’s cave – and compared our findings with the results from Härtel’s original study.

**Results:**

The optimal entry point was located at 2 mm below the horizontal plane through the angle of the mouth and just in front of the anterior edge of the mandibular ramus. From this entry point—situated around 10 mm below Härtel’s preferred entry point—Meckel’s cave was easily accessible through the medial part of FO in 17 of 22 sides.

**Conclusion:**

The findings from this study suggest that the technical results of transoval glycerol injection can be improved if we 1. Select the optimal entry point, 2. Guide the needle under fluoroscopy through the medial part of the foramen ovale, and 3. Minimize movement of the soft tissues in the cheek.

**Supplementary Information:**

The online version contains supplementary material available at 10.1007/s00701-025-06526-3.

## Introduction

Transoval glycerol injection (GI) into Meckel’s cave was introduced more than forty years ago by Håkanson as a percutaneous treatment for trigeminal neuralgia. Håkanson used fluoroscopy to guide the needle through the medial part of the foramen ovale (FO) and hit Meckel’s cave—confirmed by drainage of cerebrospinal fluid and thereafter cisternography. Otherwise, his technique was “as originally described by Härtel” [4, 5].

### Härtel’s technique

The first transoval injections for trigeminal neuralgia, into the trigeminal ganglion or sensory root, were done in 1909 with a lateral approach, just below the zygomatic arch [6, 16]. However, it was not always feasible to reach the FO and trigeminal ganglion from that angle.

In 1912, Härtel was the first to describe systematically, in two seminal papers, a direct and more reliable trajectory to the trigeminal ganglion, between the maxilla and the mandible, through the FO [7, 8].

**Fig. 1:**
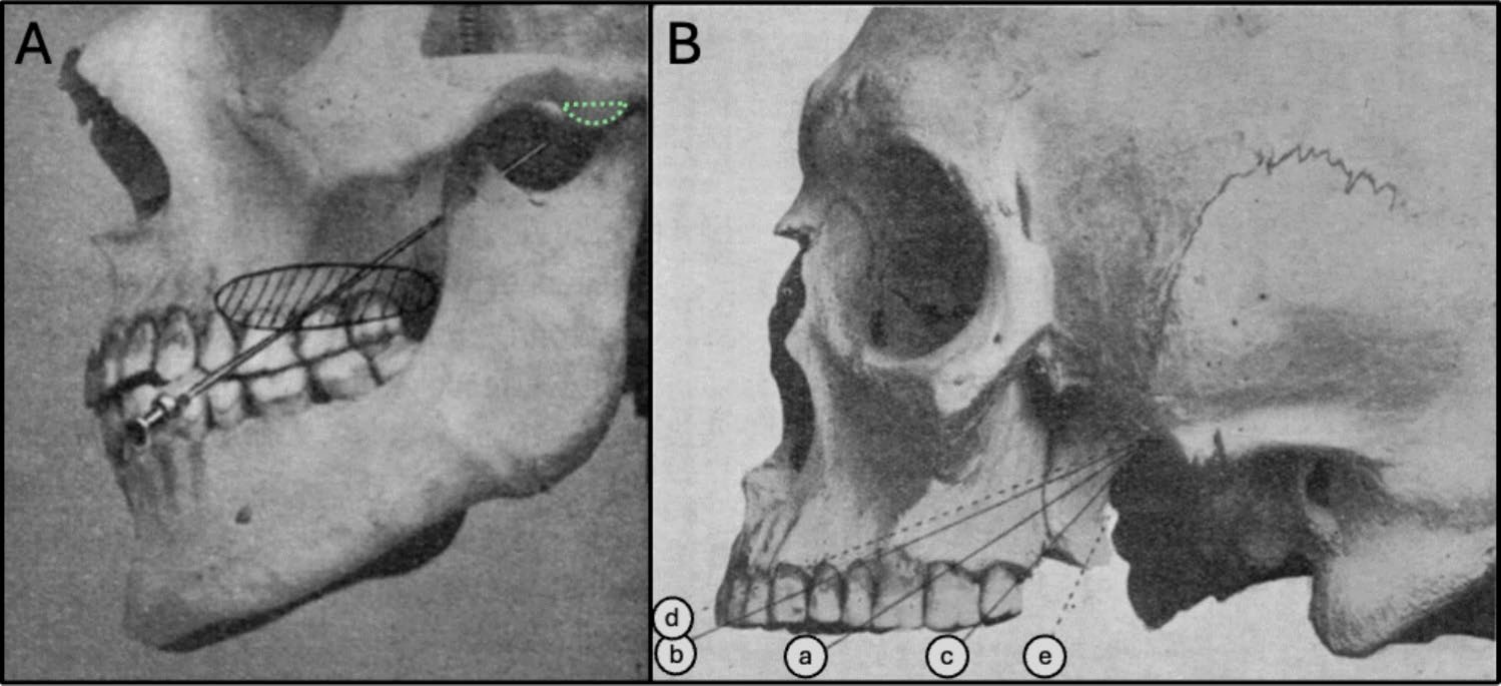
Härtel´s illustrations [7]. The letter markers a-e are modified for clarification, and the tuberculum articulare is annotated with a green dashed line. **A** Primary entry point at the base of the second upper molar, anaesthetized skin in the hatched area around in case the primary entry point failed. **B** Various trajectories discussed in Härtel’s publication, the most common is named “a”, crossing the second upper molar, while our optimal trajectory turned out to be more like “c”

In his cadaver studies, Härtel observed that the FO was not just a hole, but more like a 1 cm long canal in the sphenoid bone. He defined a safe trigeminal axis through the FO – to avoid injury to the internal carotid artery and the cavernous sinus—as starting centrally in the trigeminal impression of the petrous bone, penetrating the middle of the FO, and exiting from the cheek at the entry point, the *Einstichpunkt*. Usually, in almost 40%, the point was located at the base of the second upper molar (projected on the cheek), i.e. 3 cm behind the corner of the mouth and about 8 mm above the horizontal plane (Fig. 1).

**Fig. 2:**
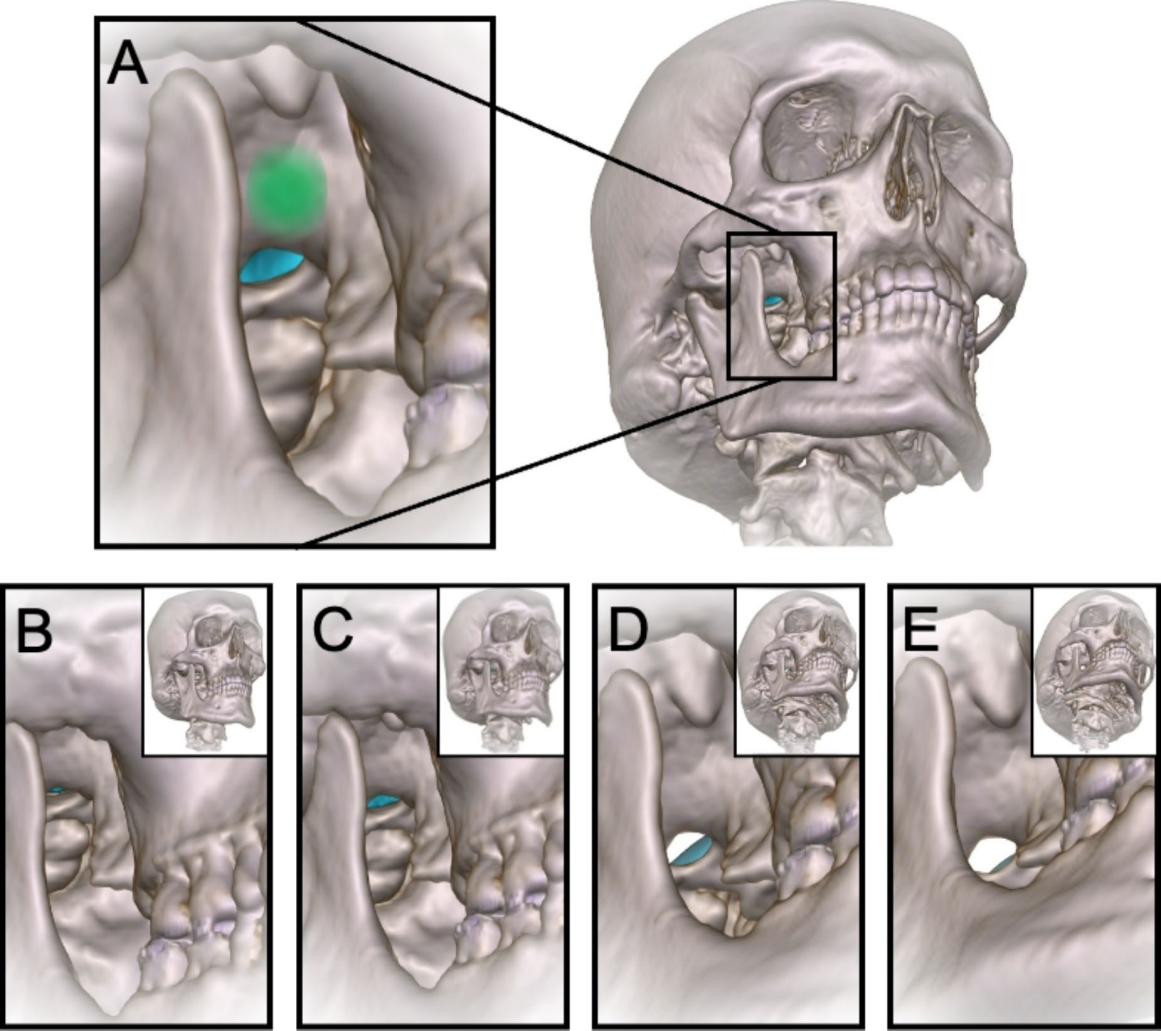
3D simulations in bone view from various angles, right side, with Meckel´s cave in blue, patient no. 8. **A** From an entry point 2 mm below the horizontal plane: Meckel’s cave is accessible through any part of the FO. The planum infratemporale is annotated in green. **B** Upper extreme entry point, closing FO. **C** Upper easy entry point, narrowing FO. **D** Lower easy entry point, Meckel’s cave beginning to hide behind posterior rim of FO. **E** Lower extreme entry point, Meckel’s cave hiding behind posterior rim of FO

From this preferred entry point in the cheek, he aimed his needle tip at two external reference points, the ipsilateral pupil, seen from the front, and the tuberculum articulare of the zygomatic arch, seen from the side (Fig. 1A). Palpating the planum infratemporale of the skull base with the needle tip, he worked his way backwards until it reached the rounded anterior medial wall of the foramen (Fig. 2A). Thereafter, the trajectory continued 1.5 cm intracranially through the FO. He used a freehand technique without fluoroscopy, inserting the needle from the Einstichpunkt in the cheek. If he failed to hit the foramen from this position, the needle was inserted just anterior to this point or just behind – at the same horizontal level (Fig. 1).

With this method, he succeeded in penetrating the foramen in 94% of the cases [8]. After alcohol injection into the ganglion, complete trigeminal anaesthesia was achieved in most of his patients, considered an optimal result. He tested the technique in 25 cases of typical and severe trigeminal neuralgia, with encouraging results at the time [8].

### Glycerol injection – a multitude of variations in technique

GI is used worldwide to relieve trigeminal neuralgia. Although Håkanson obtained excellent outcomes in his first series [5], results have been variable in later studies [10]. As Linderoth and Lind have pointed out, many neurosurgeons have created their own versions of the original technique to inject glycerol, which may explain the striking variability in the published results in comparison with the other percutaneous methods [2, 10, 15].

We think that this variability in the technical details may explain most of the poor results. We also know that the procedure is fraught with technical problems, as detailed by Blomstedt and Bergenheim in 2002 [1]. The authors met technical difficulties in half of the procedures, had to reposition the needle in more than 40% (mostly due to subtemporal needle position) and had to interrupt 8% of their procedures.

It is possible to improve the technical success rate with the use of sophisticated techniques like neuronavigation or stereotaxy, but these methods are expensive and time-consuming [17, 18].

We believe that the procedure should be simple and that a technically successful single-needle puncture of Meckel´s cave (not only FO cannulation), followed by glycerol injection, will make the procedure less painful, safer and more efficient, with minimal radiation.

### Objective

The present study therefore examines how CT- and MRI-based 3D simulation can be used to identify an optimal entry point in the cheek and provide guidance for a trajectory through FO to reach the trigeminal cistern. In addition, we use simulation to define Härtel’s trigeminal axis and the Einstichpunkt—thus imitating his study on a small scale for comparison.

## Methods

### Coregistration and segmentation of imaging data

Eleven patients (5 women, 6 men, mean age 60 years) were included in this study. They had been referred for neurosurgical treatment of trigeminal neuralgia. The main inclusion criteria were available routine CT that included the skull base and mandible in its field of view and MRI with thin T2-weighted series, such as “CISS”, “T2 space”, or similar, through the trigeminal nerve area with Meckel’s cave included in its entirety. Both the CT and MRI images had to have less than or equal to 1 mm thick slices for good 3D visualizations, otherwise, the protocols were not standardized and scans from several different locations were included. The CT and MRI scans were coregistered using 3D Slicer software (available at http://www.slicer.org) [9]. The coregistration process involved landmark registration, where we identified and placed points on the semicircular canals, visible in both MRI and CT scans, to achieve satisfactory alignment. Meckel's caves were segmented on the coregistered MRI T2 images by a radiologist. Further visualization and measurements were also done using 3D Slicer.

### Visualization techniques

**Fig. 3:**
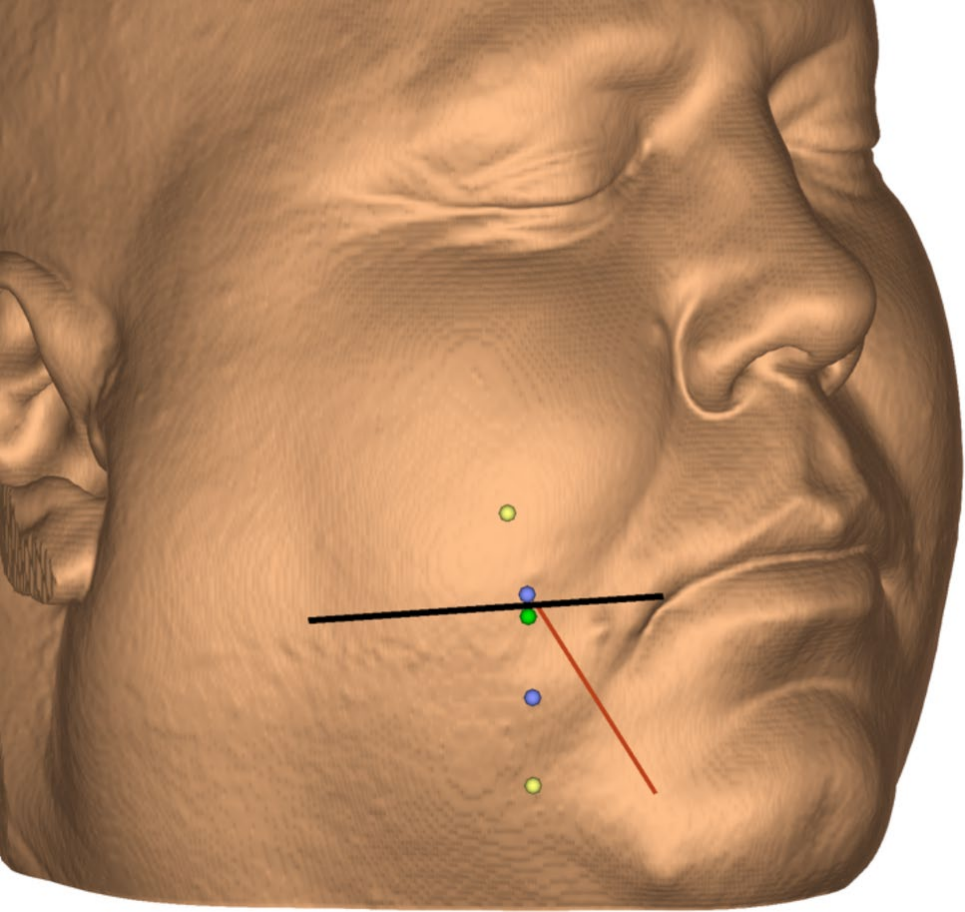
Skin view visualization of an illustrative case with annotations (patient no. 8). Horizontal line in black indicates where the horizontal plane intersects the cheek. Upper and lower entry point extremes in yellow. Upper and lower boundaries for “easy hits” in blue. Our optimal entry point at 2 mm below the horizontal in green. Simulated Härtel’s trigeminal axis in red, in this case exiting at the horizontal level

For precise visualization, we employed two types of 3D volume rendering of the CT scans:

1. Bone view: To observe the mandible, the FO and the segmented Meckel's cave (Fig. 2).
2. Skin view: This setting was used for point placement on the face (Fig. 3).

### Determination of needle insertion points

We simulated a trajectory from the entry point in the cheek through the FO towards Meckel’s cave – parallel to the angle of view.

1. Tracing along the anterior border of the mandibular ramus, we simulated two entry points—the upper and lower extremes in the cheek from where Meckel’s cave could *possibly* be accessed through the FO (Fig. 2B and E).
2. We also defined a span for easy entry points (“easy hits”)—between the highest and lowest level on the cheek from where Meckel’s cave was *easily* accessible through half or more of a distinct FO (Fig. 2C and D)—and laterally, close to the anterior edge of the mandibular ramus, to expose more of Meckel´s cave in the foramen.
3. “Possible hits” were defined as entry points between the upper and lower extremes and outside the easy entry point span.
4. Thereafter, we simulated Härtel’s trigeminal axis for all 22 foramina—from the midpoint of the trigeminal impression through the central part of the FO and then penetrating the cheek—to obtain the coordinates of the Einstichpunkt (Figs. 2 and 3).

### Measurements and calculations

The entry points’ coordinates were measured from a plane termed “the horizontal plane through the angle of the mouth”, defined as the functional occlusive plane between the teeth [11]. We recorded the coordinates of the upper and lower points in millimetres, noting whether these were above (positive values) or below (negative values) the reference horizontal plane.

## Results

Simulating the trajectory from below, we found a total span—between the upper and the lower extreme entry points—varying between 17 mm (in a patient with a narrow FO) and 58 mm (in two obese patients), with an average of 35 mm. Through all 22 foramina, Meckel’s cave could possibly be reached from an entry point located around 3 cm laterally from the corner of the mouth and 1–7 mm below the horizontal level (Figs. 2 and 5).

Spans for easy entry points (wide open FO with easily accessible Meckel’s cave) were between 5 and 47 mm wide, on average 18 mm, mostly located between − 25 and + 25 mm, all intersecting the interval between + 5 and − 8 mm (Fig. 5).

**Fig. 4:**
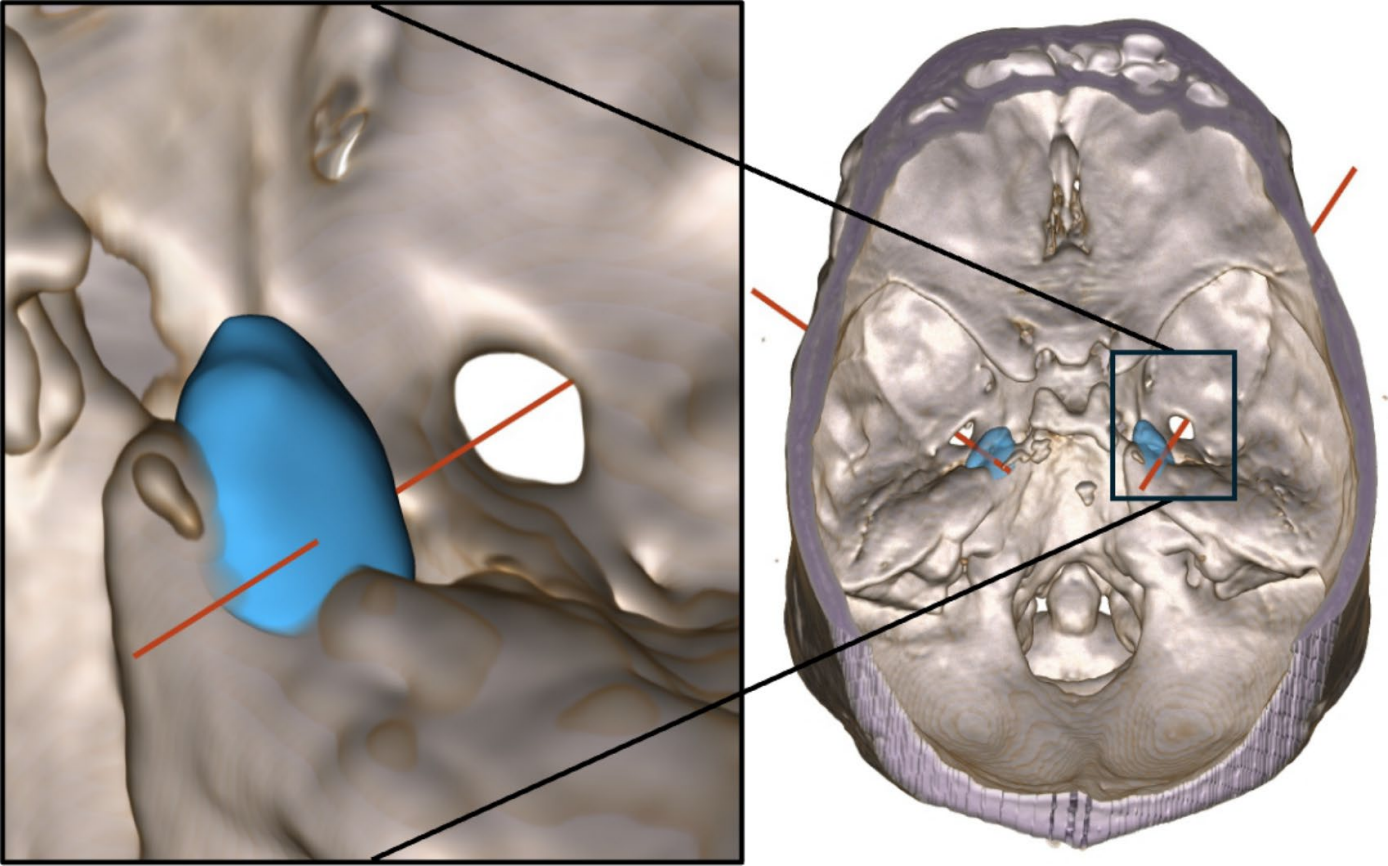
Visualization of patient no. 8, right side, from inside the skull. Meckel’s cave in blue. Simulated Härtel’s trigeminal axis in red from the centre of the trigeminal impression, through Meckel’s cave, and through FO centre. The zoomed-in image has a slightly different rotation than the overview image to better highlight the relationship between the FO and Meckel’s cave

**Fig. 5:**
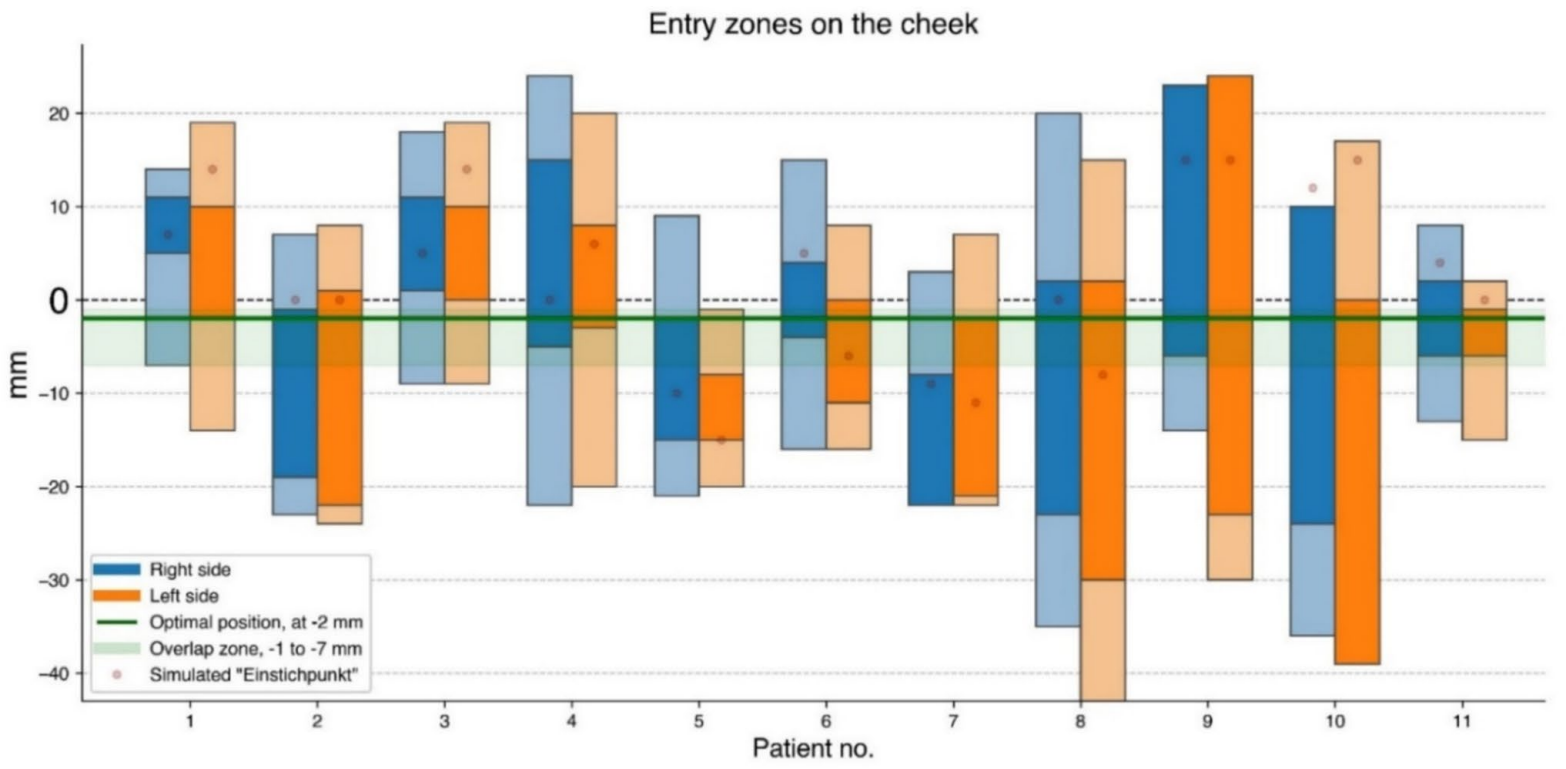
Spans between upper and lower entry points in the cheek. Lighter colours represent spans between extreme entry points; darker colours represent spans with easy entry points. Simulated Einstichpunkte (Härtel’s) are marked with a point

Härtel’s trajectories, the trigeminal axes, simulated from above (from the trigeminal impression through FO towards the cheek), exited from the cheek at various Einstichpunkte scattered at a horizontal level between + 15 mm and − 15 mm, on the average 2.4 mm (median 2 mm) above zero level (Figs. 4 and 5). If we elevated the simulated needle tip 1–2 mm above the middle of the trigeminal impression, still inside Meckel’s cave, the trajectory exited 4–8 mm lower on the cheek.

**Fig. 6:**
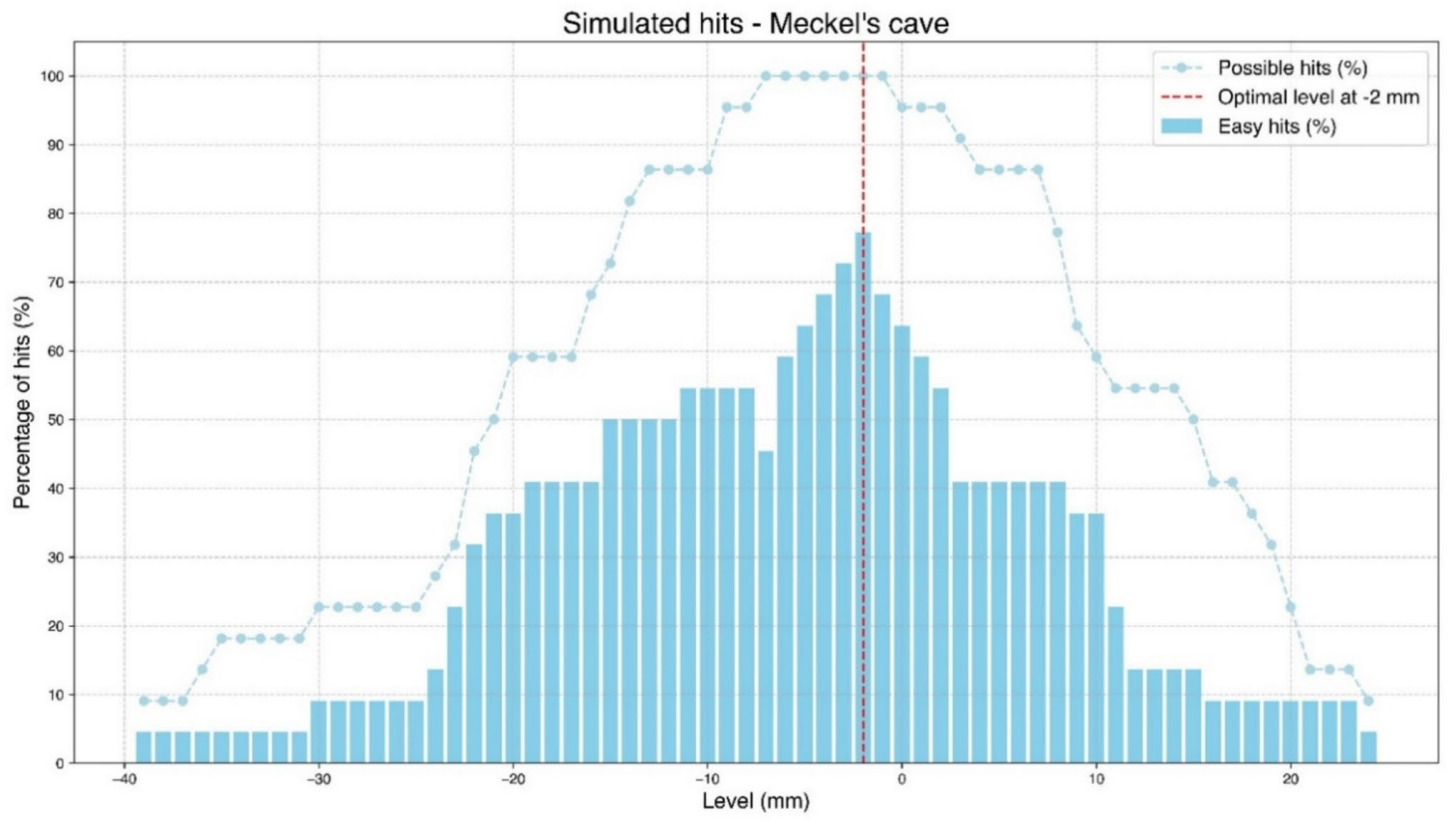
Percentage of hits in 22 foramina at horizontal levels between − 39 mm and + 24 mm

At the best level in our simulations, 2 mm below the horizontal plane, Meckel’s cave was easily accessible through the medial part of FO in 17 of 22 foramina (Figs. 5 and 6). In the remaining five FOs, at the − 2 level, Meckel’s cave was not easily accessible, but could be reached by keeping the needle as medially as possible in all the five FOs (Supplementary Fig. 1).

Just above the horizontal level, at + 5 mm, eight of the FOs presented as narrow slitlike projections, difficult to access. With higher entry points, above + 10 mm, most (16 of 22) of the foraminal openings became slitlike, therefore more difficult to penetrate (Figs. 2B and 5).

With a lower entry point in the cheek, just below − 2 mm, the FO opened up widely. However, below − 7 mm, the posterior rim covered most of Meckel´s cave in three of the FOs. A little bit lower, at − 10 mm, three more of the 22 Meckel’s caves were inaccessible (Fig. 5).

### Clinical examples

**Fig. 7:**
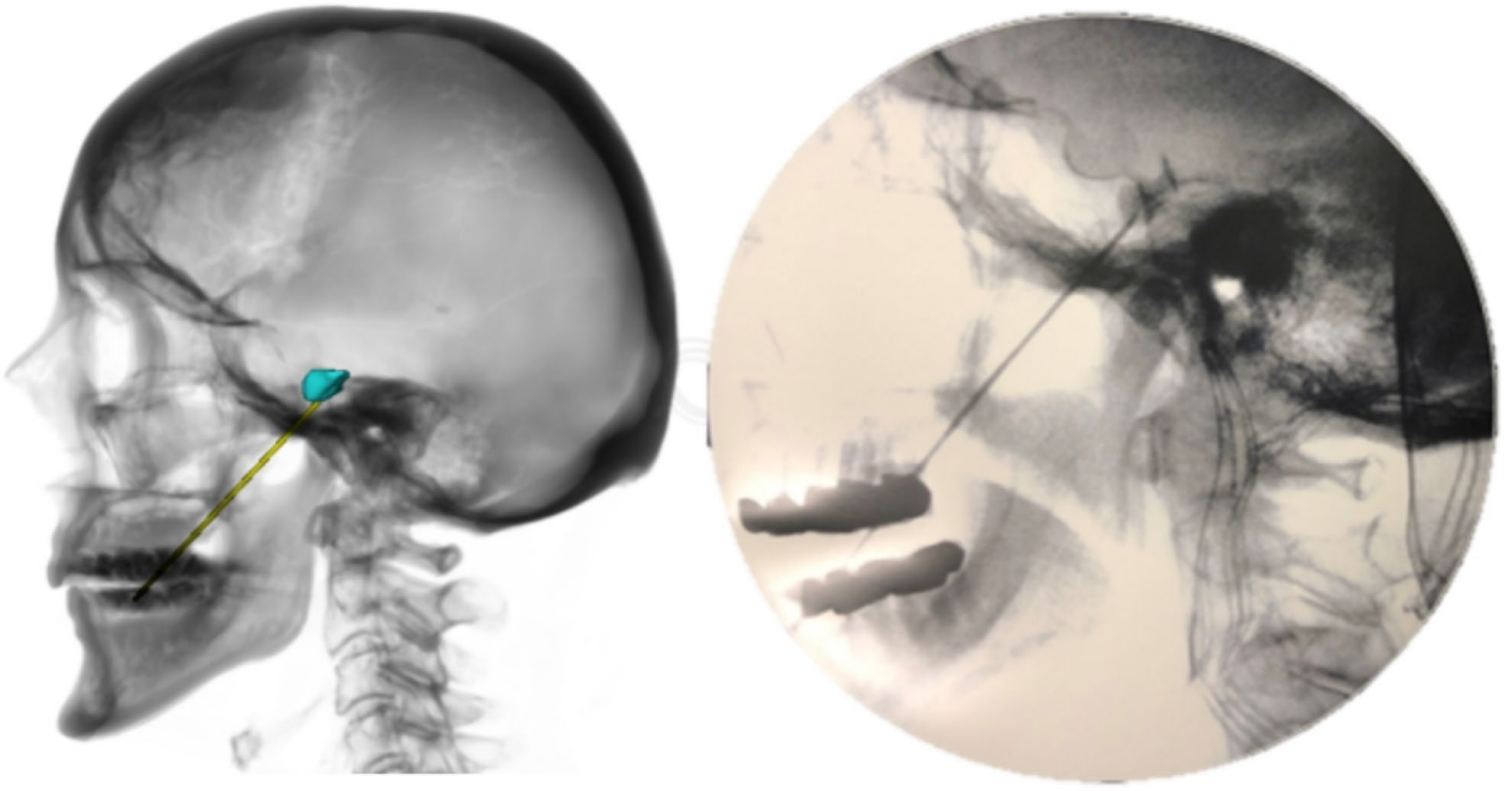
Left: Preoperative simulation of trajectory and cistern. Right: Cisternography in the same patient

Three of the patients were treated with glycerol injections after simulation – with an entry point in the lower part of the easy entry span – all were successful primary hits. One example is shown in Fig. 7. Five other patients had already been treated, four were primary hits with entry points inside the optimal span, and one was a failure with subtemporal needle position – where later simulation indicated too anterior puncture of FO – missing Meckel’s cave. All seven cisternographies were typical and corresponded well with our 3D simulations.

## Discussion

In the present imaging-based study of 11 patients referred for trigeminal neuralgia, we failed to identify a single ideal entry point in the cheek for transoval puncture of Meckel’s cave. This is in keeping with findings in Härtel’s original cadaver study where he observed that the Einstichpunkt’s position for transoval trigeminal injections varied between individuals, and was located in the region of the upper three molars, as indicated in Fig. 1A [7]. Our observation also corresponds with Peris-Celda et al.’s anatomical study showing that one ideal entry point could not be established, and concluding that the needle should be inserted at the horizontal level—somewhere in the wide range between 10 and 35 mm (on average 25 mm) lateral to the angle of the mouth [12].

Nevertheless, our simulation identified an optimal entry point in the cheek, 2 mm below the horizontal level and around 3 cm lateral to the angle of the mouth, just in front of the anterior edge of the ramus mandibulae. Using this entry point in our simulations, we observed that Meckel’s cave could be easily hit through the medial part of the foramen in 17 of the 22 simulations (77%). The remaining five targets could theoretically be reached from the same entry point by keeping the needle close to the medial rim of the FO, alternatively by lowering or elevating the entry point—in the interval between + 5 and − 8 mm (Supplementary figure). In the present study, our simulated optimal entry point with at least 77% hits was situated around 10 mm below Härtel’s best Einstichpunkt at the base of the second upper molar, from where nearly 40% of the foramina were punctured in his study [7]. This apparent discrepancy may in part be explained by differing targets. Our target was any point well inside Meckel’s cave accessible through the medial part of the FO—which is different from Härtel’s central point in the trigeminal impression. Our simulated trajectory was therefore more medial and slightly steeper than Härtel’s trigeminal axis—indicating that our trajectory was 1–2 mm higher at the level of the trigeminal impression – which in turn explains the lower entry point identified in our simulation (Fig. 1). It is also probable that some of Härtel’s specimens in the cadaver study were cachectic with hollow cheeks (Fig. 15 and 16 in [7]) – resulting in a higher Einstichpunkt than in our imaging study of living individuals.

Our findings correspond well with Peris-Celda & al.’s cadaver study [12]. They inserted a needle from the medial part of the trigeminal impression towards the medial part of the FO – comparable to our simulation—and found an exit point in the cheek on average 3.6 mm below the oral commissure, varying between 7 mm above and 15 mm below.

We simulated Härtel’s trigeminal axis and found, on average, its exit in the cheek at the + 2.4 mm level, around 5 mm below Härtel’s preferred Einstichpunkt—but our results were widely scattered at horizontal levels between + 15 mm and – 15 mm. The methods were different. Härtel used a 0.8 mm wide cannula inserted with a freehand technique from the trigeminal impression through FO in cadavers, while we traced a virtual line in 3D images from patients with trigeminal neuralgia. Härtel’s study is impressive with 61 cadaver skulls examined, but we tend to believe that our small study gives a more accurate spatial determination of the trigeminal axis.

In our clinical experience, the needle tip will move a few mm upwards just after insertion into the cheek, probably due to its direction through the loose soft tissue and to facial muscular contractions in the awake patient. To compensate for this, we think that the optimal entry point in clinical practice will be situated a few mm lower on the cheek than the − 2 mm level observed in our simulations. Our previous standard entry point in the cheek, at the horizontal level, resulted in technically successful procedures in nearly 80% of patients with one single needle puncture medially in the FO [14], but the success rate increased to above 90% after we lowered the entry point to − 10 mm. This improvement may be explained by our findings in the present study—if we assume that the soft tissues are moved five mm upwards during the procedure.

Technical problems are common in percutaneous GI. Many of them can be overcome by adhering to strict procedural techniques. Our present findings suggest an optimal needle insertion point just below the horizontal level and just anterior to the mandibular ramus – mostly in keeping with Linderoth et al.’s recommendations (horizontal level, 3–4 cm lateral to the corner of the mouth, medial part of the FO) [10]. 3D simulations of CT with Meckel’s cave segmentations may be helpful in difficult cases since anatomical variations or anomalies are frequent [3].

### Limitations and strengths

This is a clinical case series where we have studied routine images in a small sample of 22 sides in 11 patients, while Härtel examined 122 foramina in 61 cadavers [7]. The selection of our patients was based on available CT and MRI scans on referral to neurosurgery. Co-registration, Meckel’s cave segmentation, simulations, and measurements were done twice to confirm that the results were reproducible. Variations were minimal (median 2 mm), and we chose to use the measurements from our second round, estimated to be the more robust, due to a learning effect.

Neither the cited cadaver studies nor our imaging study have paid much attention to the variability and movability of the soft tissues. In cadavers, soft tissues will be stiff and shrunk due to formalin fixation and loss of body fluids. Premortal and postmortal changes will probably be more pronounced in the cheek than around the trigeminal ganglion. Hollow cheeks in cachectic specimens might disturb the measurements of entry point coordinates in the cheek, a source of error in cadaveric studies. Rubinstein & al. have shown that intracranial trigeminal anatomy in cadaver specimens closely resembles findings in imaging studies [13]. We therefore believe that our small study with simulation-based upon living anatomy may have an advantage and can be compared to Härtel’s and Peris-Celda’s studies. Movability and volume of the soft tissues should nevertheless be considered when transoval injections are used in patients with trigeminal neuralgia.

To our knowledge, this is the first imaging-based study to determine the most reliable transoval route to Meckel’s cave in a sample of patients referred for neurosurgery. Our conclusions from the simulations have not been evaluated systematically in the treatment of patients, but are in keeping with our clinical experience.

## Conclusion

More than a hundred years ago, Härtel presented a pioneering technique for transoval alcohol injections into the trigeminal ganglion [7, 8]. His approach is still used worldwide in ablative percutaneous treatments of trigeminal neuralgia directed at the ganglion or the trigeminal nerve. Härtel identified a primary puncture site in the cheek, the average Einstichpunkt, at the base of the second upper molar, i.e. around 8 mm above the horizontal plane through the oral commissure. Our findings in the present study suggest that the technical success rate for transoval glycerol injections into Meckel’s cave will increase if we select a lower entry point in the cheek, i.e. 2 mm below the horizontal plane (assuming that the soft tissues are immovable) and, under fluoroscopy, aim at the medial part of the foramen ovale. In difficult cases, such as anatomical variations or anomalies, preoperative 3D simulation may be helpful.

## Supplementary Information

Below is the link to the electronic supplementary material.

Supplementary file1 (DOCX 337 KB)

## Data Availability

No datasets were generated or analysed during the current study.

## Abbreviations

FO: Foramen ovale
GI: Percutaneous transoval glycerol injection
MRI: Magnetic resonance imaging
CT: Computerized tomography

## References

[1] Blomstedt PC, Bergenheim AT (2002) Technical difficulties and perioperative complications of retrogasserian glycerol rhizotomy for trigeminal neuralgia. Stereot Funct Neuros 79:168–181. 10.1159/000070830 10.1159/000070830 12890975

[2] Chang KW, Jung HH, Chang JW (2022) Percutaneous procedures for trigeminal neuralgia. J Korean Neurosurg Soc 65:622–632 35678088 10.3340/jkns.2022.0074 PMC9452389

[3] Elnashar A, Patel SK, Kurbanov A, Zvereva K, Keller JT, Grande AW (2020) Comprehensive anatomy of the foramen ovale critical to percutaneous stereotactic radiofrequency rhizotomy: cadaveric study of dry skulls. J Neurosurg 132:1414–1422. 10.3171/2019.1.JNS18899 31003215 10.3171/2019.1.JNS18899

[4] Hakanson S (1978) Transoval trigeminal cisternography. Surg Neurol 10:137–144 705591

[5] Hakanson S (1981) Trigeminal neuralgia treated by the injection of glycerol into the trigeminal cistern. Neurosurgery 9:638–646. 10.1227/00006123-198112000-00005 7322329 10.1227/00006123-198112000-00005

[6] Harris W (1909) The alcohol injection treatment of neuralgia and spasm. Proc R Soc Med 2:77–91 19973797 10.1177/003591570900200717 PMC2046550

[7] Härtel F (1913) Die Leitungsanästhesie und Injections-behandlung des Ganglion Gasseri und der Trigeminusstämme. In: Härtel F (ed) Die Leitungsanästhesie und Injections-behandlung des Ganglion Gasseri und der Trigeminusstämme. Springer Berlin Heidelberg, Berlin, pp 1–100. 10.1007/978-3-662-26170-5_1

[8] Härtel F (1914) Die Behandlung der Trigeminusneuralgie mit intrakraniellen Alkoholeinspritzungen. Deutsche Zeitschrift Chirurgie 126:429–552. 10.1007/BF02800919

[9] Kikinis R, Pieper SD, Vosburgh KG (2014) 3D Slicer: a platform for subject-specific image analysis, visualization, and clinical support. In: Jolesz FA (ed) Intraoperative imaging and image-guided therapy. Springer New York, New York, pp 277–289. 10.1007/978-1-4614-7657-3_19

[10] Linderoth B, Lind G (2009) Retrogasserian glycerol injection for trigeminal neuralgia. In: Lozano AM, Gildenberg PL, Tasker RR (eds) Textbook of stereotactic and functional neurosurgery. Springer Berlin Heidelberg, Berlin, pp 2429–2456. 10.1007/978-3-540-69960-6_142

[11] Madsen DP, Sampson WJ, Townsend GC (2008) Craniofacial reference plane variation and natural head position. Eur J Orthod 30:532–540. 10.1093/ejo/cjn031 18632837 10.1093/ejo/cjn031

[12] Peris-Celda M, Graziano F, Russo V, Mericle RA, Ulm AJ (2013) Foramen ovale puncture, lesioning accuracy, and avoiding complications: microsurgical anatomy study with clinical implications. J Neurosurg 119:1176–1193 23600929 10.3171/2013.1.JNS12743

[13] Rubinstein D, Stears RL, Stears JC (1994) Trigeminal nerve and ganglion in the Meckel cave: appearance at CT and MR imaging. Radiology 193:155–159. 10.1148/radiology.193.1.8090884 8090884 10.1148/radiology.193.1.8090884

[14] Slettebø H, Hirschberg H, Lindegaard K-F (1993) Long-term results after percutaneous retrogasserian glycerol rhizotomy in patients with trigeminal neuralgia. Acta Neurochir 122:231–235 8372713 10.1007/BF01405534

[15] Staudt MD, Rivera M, Miller JP (2018) Percutaneous procedures for trigeminal neuralgia. Diagnosis and management of head and face pain: a practical approach. pp 221–233. 10.1007/978-3-319-90999-8_18

[16] Taptas N (1911) Les injections d’alcool dans le ganglion de Gasser a travers le trou ovale. Presse méd 19:798

[17] Wang Z, Su X, Yu Y, Wang Z, Li K, Gao Y, Tian Y, Du C (2022) A review of literature and meta-analysis of one-puncture success rate in radiofrequency thermocoagulation with different guidance techniques for trigeminal neuralgia. Eur J Med Res 27:141. 10.1186/s40001-022-00758-0 35933404 10.1186/s40001-022-00758-0 PMC9356501

[18] Wu FHW, Cheung CW, Leung YY (2024) Neuronavigation-guided percutaneous rhizotomies to trigeminal neuralgia: a systematic review. Clin J Pain 40:253–266. 10.1097/AJP.0000000000001191 38193245 10.1097/AJP.0000000000001191

