# Supplementary material for: Revisiting Härtel’s technique for percutaneous transoval glycerol injection
Source: Acta Neurochir (Wien). 2025 Apr 30;167(1):126. doi: 10.1007/s00701-025-06526-3 (PMC12043756; doi:10.1007/s00701-025-06526-3)
Supplement: Supplementary file 1 — Supplementary file1 (DOCX 337 KB) [file 701_2025_6526_MOESM1_ESM.docx]

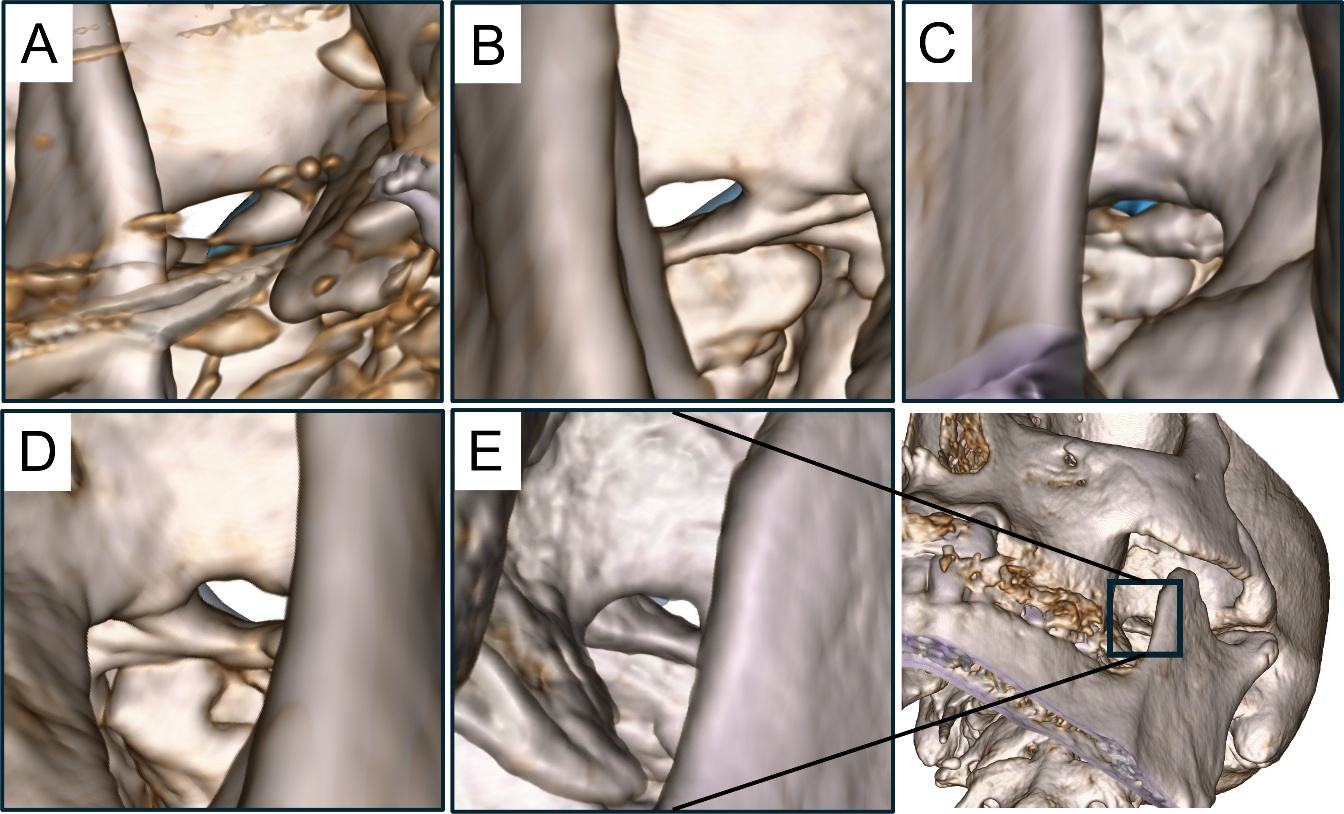


**Supplementary Fig. 1** Bone view simulations from the -2 mm horizontal level of the five sides where Meckel’s cave could possibly be hit, but not easily. **A-C** right sides, **D, E** left sides.
